# A new approach to left sleeve pneumonectomy: complete VATS left pneumonectomy followed by right thoracotomy for carinal resection and reconstruction

**DOI:** 10.1186/s40792-018-0496-2

**Published:** 2018-08-10

**Authors:** Toshio Fujino, Masayuki Tanahashi, Haruhiro Yukiue, Eriko Suzuki, Naoko Yoshii, Masayuki Shitara, Yasunori Kaminuma, Hiroshi Niwa

**Affiliations:** Division of Thoracic Surgery, Respiratory Disease Center, Seirei Mikatahara General Hospital, Hamamatsu, Japan

**Keywords:** Left sleeve pneumonectomy, New approach, Tracheobronchial tumor

## Abstract

**Background:**

Left sleeve pneumonectomy is a challenging operation that requires individualized approaches. Here, we present a new minimally invasive combined thoracoscopic approach.

**Case presentation:**

A 61-year-old woman was diagnosed with tracheobronchial adenoid cystic carcinoma. The tumor originated from the left main stem bronchus, and tumor with carinal involvement was observed. We judged that complete resection would be possible via left sleeve pneumonectomy. However, because tumor involvement with the esophagus and descending aorta was suspected, evaluation of resectability in advance was necessary. After confirmation via examination thoracoscopy of no involvement with the surrounding organs, complete VATS left pneumonectomy was performed and followed by right thoracotomy for carinal resection and reconstruction.

**Conclusions:**

When thoracoscopic surgery becomes mainstream, this minimally invasive combined thoracoscopic approach might be an optimal option for patients who require left sleeve pneumonectomy.

## Background

Several approaches to left sleeve pneumonectomy (LSP) have been reported owing to anatomical restrictions in the left thoracic cavity. In addition to bilateral thoracotomy, left thoracotomy alone and median sternotomy, the clamshell approach has recently been reported as useful. Because each approach has advantages and disadvantages, treatments should be individualized [1]. As thoracoscopic surgery is becoming more mainstream, new, less invasive approaches to extended surgery, such as LSP, are required. Here, we present a new minimally invasive combined thoracoscopic approach and its benefits.

## Case presentation

**Fig. 1:**
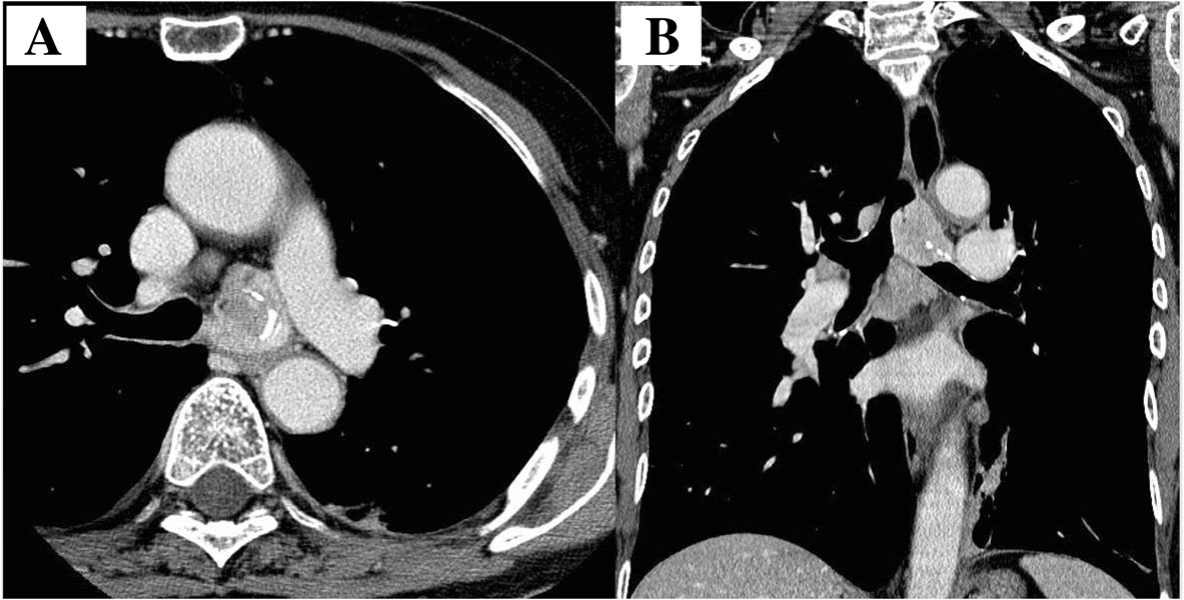
Computed tomography findings. **a** Coronal and **b** Sagittal view. A solid left main bronchial tumor with carinal involvement is observed

**Fig. 2:**
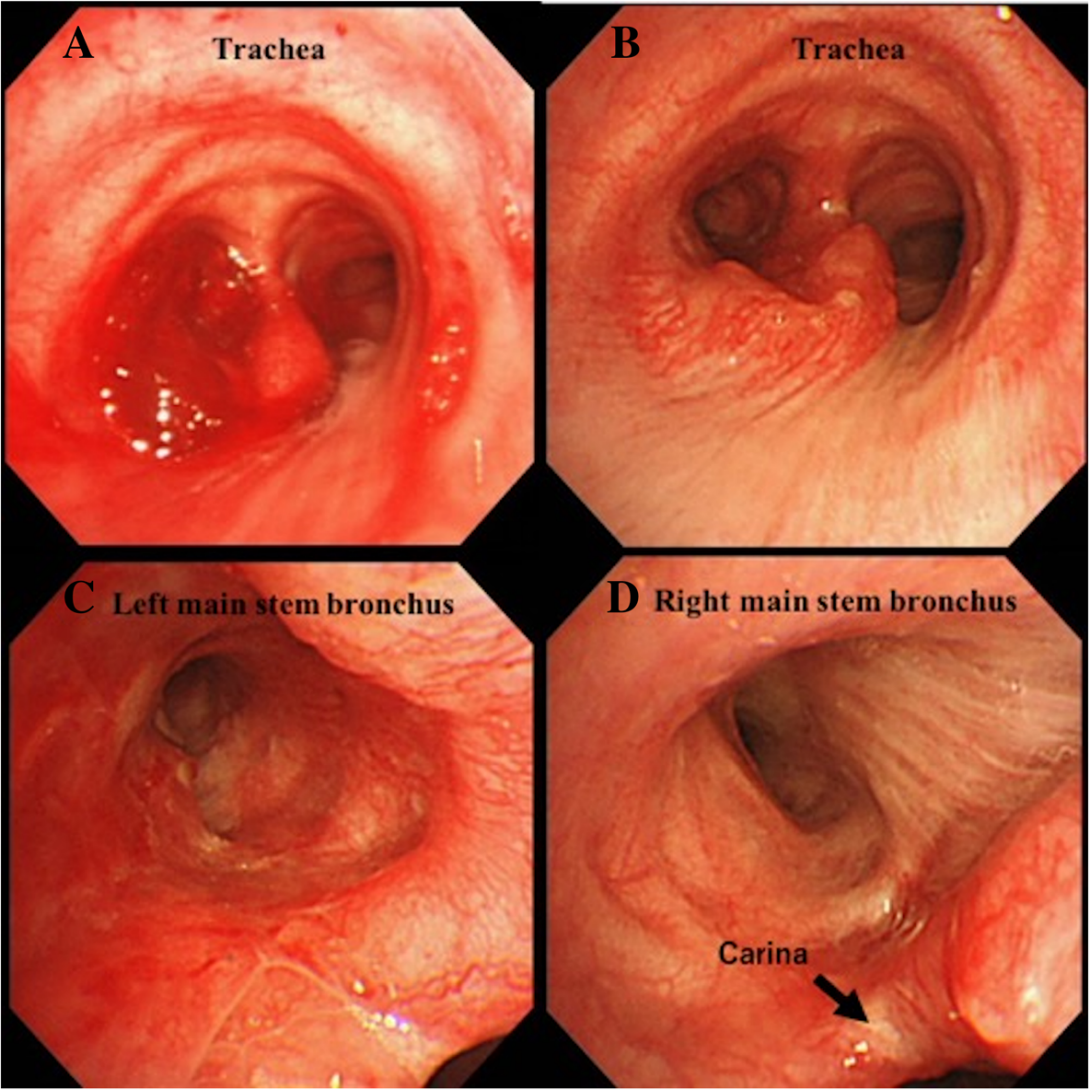
Endoscopic findings. **a** Before rigid bronchoscopic treatment. The tumor almost completely occludes the left main stem bronchus. **b**, **c**, and **d** Bronchoscopy 2 weeks after rigid bronchoscopic treatment. Almost the entire left main stem bronchus, two tracheal cartilage rings above the carina, and one right main stem bronchial ring distal from the carina are invaded by the tumor

**Fig. 3:**
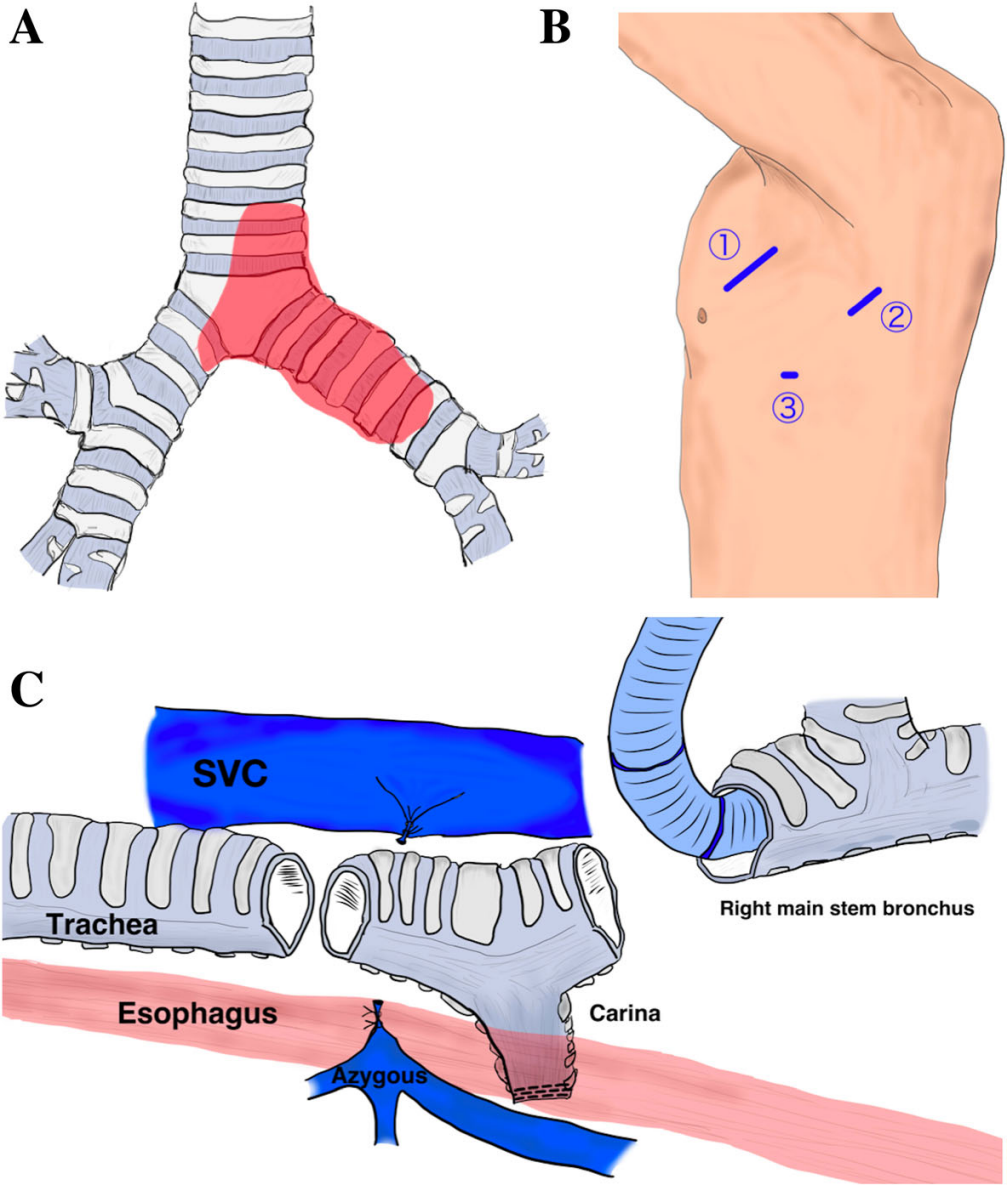
Schematic figures. **a** Tumor location. **b** c-VATS establishment: ①Access window, 4th intercostal space, 50 mm; ②Assist window, 6th intercostal space, 40 mm; ③Camera port, 7th intercostal space, 12 mm. **c** Posterolateral thoracotomy, the carinal resection and reconstruction were completed under surgical field intubation

A 61-year-old woman was seen in a clinic complaining of fever and dyspnea lasting 2 weeks. She was diagnosed with left pneumonia by computed tomography (CT) and referred to our institution. At the time of presentation, she had dyspnea and a fever over 38 °C. Her Eastern Cooperative Oncology Group (ECOG) performance status score was 2, and her Hugh-Jones classification was IV. Rhonchi were evident in the anterior chest. Laboratory studies showed an increased inflammatory response. All tumor markers were within normal limits. The chest X-ray revealed an infiltrative shadow in the lower left lung field. CT imaging showed a solid left main bronchial tumor with carinal involvement. Cartilage destruction was apparent, and the boundaries between the tumor and the esophagus and descending aorta were unclear (Fig. 1). Therefore, tumor infiltration into the esophagus and descending aorta was suspected. We diagnosed her with obstructive pneumonia due to a tracheobronchial tumor. For the purposes of securing the airway, performing a tissue diagnosis and evaluating the extent of tumor progression, rigid bronchoscopy was initially performed. The tumor almost completely occluded the left main bronchus, and tumor hemorrhage was evident. By coring out the tumor, the left main bronchus was reopened, and detail of the involved area was revealed. The tumor originated from the left main stem bronchus and occupied almost the entire left main stem bronchus. Two tracheal cartilage rings above the carina and one right main stem bronchial ring distal from the carina were invaded by the tumor (Figs. 2 and 3a). The pathological examination showed three typical types of histology (cribriform, tubular, solid pattern), and she was diagnosed with tracheobronchial adenoid cystic carcinoma. The rigid bronchoscopic treatment resulted in a significant improvement in the patient’s general condition and cardiopulmonary function (PS 2 → 0, H-J IV → I). Fluorodeoxyglucose-positron emission tomography (FDG-PET) showed abnormal enhancement in the tumor with a maximum standard uptake value of 5.3. At the other sites, abnormal FDG uptake was not observed. We determined that if the tumor did not infiltrate the surrounding organs, complete resection would be possible via LSP. Even if a microscopic lesion remains in the resected stump, we can expect improvement in prognosis by administering additional postoperative irradiation. Therefore, we decided to perform the surgery.

**Fig. 4:**
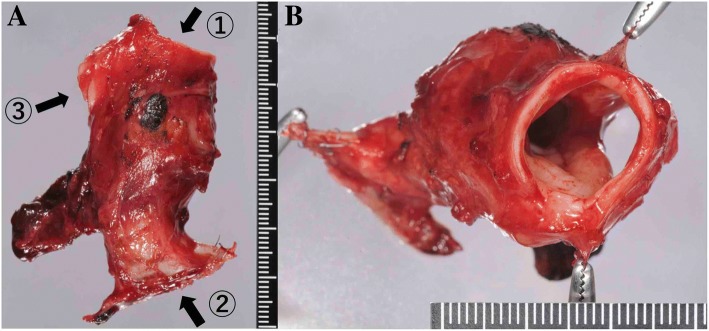
Macroscopic findings of the resected tracheobronchial tumor. **a** A front view of the ①tracheal stump, ②left main bronchial stump, ③right main bronchial stump. **b** The tumor and carina are observed from the tracheal lumen

To evaluate the presence of out-of-wall invasion, we proceeded with the left-side operation in the right lateral decubitus position by complete VATS with three ports in a look-up setting (Fig. 3b). A right-sided double lumen endotracheal tube was used. Initially, the pleura was opened along the subaortic window. We observed no tumor infiltration into the left recurrent laryngeal nerve, esophagus, or descending aorta. Next, we performed a left pneumonectomy by complete VATS. The left pulmonary artery and vein were dissected into their extrapericardial sections by staplers. Subsequently, the left main stem bronchus was cut near the second carina (where the tumor had not progressed) using the stapler. A protective bag provided easy removal of the left lung from the pleural cavity without enlarging the skin incision. After that, the tracheal carina was exposed circumferentially, and the lower trachea and right main stem bronchus were identified by forceps manipulation. The left chest was closed. After exchanging for a 7.5-Fr single-lumen endotracheal tube, the surgical position and approach were switched to the left lateral decubitus position with a posterior lateral incision and a fourth intercostal thoracotomy. Low volume ventilation with small expansion of the right lung simplifies the operation. Prior identification via left thoracotomy around the carina provided easy circumferential exposure of the trachea, carina, and right main stem bronchus after dissecting the azygous vein. Then, the right main stem bronchus was dissected at the two rings distal from the carina followed by surgical field intubation using a 6.5-Fr spiral tube with a short cuff. The tracheal carina was removed after dissection of the three rings above the carina (Figs. 3c and 4). Under surgical intubation, the trachea and right main stem bronchus were anastomosed with a telescope technique using interrupted sutures with full-thickness bites and 4-0 PDS. After completing a left-side semicircle anastomosis by surgical intubation, a right-side semicircle anastomosis was performed under intermittent removal of the tube after sufficient oxygenation during one or two stiches. There was no requirement for prepared jet ventilation. The anastomotic site was wrapped with the intercostal muscle pedicles, and the operation was terminated. To avoid tension on the anatomic site, the chin was tagged to the anterior chest wall by two sutures for 2 weeks. Pathological analysis revealed no tumor component in the resected stump, and we achieved complete resection. After the operation, the patient experienced a panic attack, and hospitalization was prolonged. She improved with psychiatric intervention. She was discharged and walking independently on postoperative day 79. Unfortunately, recurrence via bone metastasis to the left humerus was observed 6 months after surgery, and palliative irradiation is underway. We are currently monitoring her progress.

LSP is one of the most challenging operations in thoracic surgery, and surgical approaches need to be individualized. Bilateral thoracotomy and median sternotomy are often favored; [2] however, as thoracoscopic surgery becomes mainstream, newer and less invasive approaches for extended surgery, such as LSP, are employed.

Cases for which LSP is indicated are generally locally advanced malignant tumors that often involve surrounding organs, and proper assessment is critical. If tumor invasion to the surrounding organs is suspected and preservation of the left lung cannot be expected upon initial diagnosis, this minimally invasive combined thoracoscopic approach has several advantages. With initial left-sided VATS, resectability can be evaluated in advance and in a less invasive manner than with thoracotomy. Confirmation of no invasion to the surrounding tissue makes left pneumonectomy beneficial. Right thoracotomy provides safety and precise anastomosis at the time of carinal reconstruction.

An initial right thoracotomy could be considered, but it is difficult to evaluate tumor involvement in the left thorax [3]. Initial right thoracotomy requires tube intubation through the narrowed left main stem bronchus with tumor invasion. It is difficult to insert a large caliber tube enough to maintain ventilation and oxygenation. In recent years, the usefulness of the clamshell approach for carinal reconstruction has been reported, [4] but it has several disadvantages, such as poor visibility of the esophagus and descending aorta and the requirement of extensive detachment of respiratory muscles. The disadvantage of poor visibility is similar in anterior approaches such as the transsternal and hemi-clamshell approaches. However, our approach requires a position change, and there are also disadvantages relative to providing ventilation during airway anastomosis, which is complicated and difficult to address in an emergency. In our case, small volume ventilation from the surgical field provided precise anastomotic maneuvering. Because of the difficulty of laryngeal release, this approach is not suitable when the resection length of the trachea is relatively long.

## Conclusions

This minimally invasive combined thoracoscopic approach might be an optimal option for patients who require left sleeve pneumonectomy.

## Abbreviations

CT: Computed tomography
FDG-PET: Fluorodeoxyglucose-positron emission tomography
LSP: Left sleeve pneumonectomy
VATS: Video-assisted thoracoscopic surgery
